# Application of CAD Systems in Breast Cancer Diagnosis Using Machine Learning Techniques: An Overview of Systematic Reviews

**DOI:** 10.3390/bioengineering12111160

**Published:** 2025-10-27

**Authors:** Theofilos Andreadis, Antonios Gasteratos, Ioannis Seimenis, Dimitrios Koulouriotis

**Affiliations:** 1 Department of Production and Management Engineering, Democritus University of Thrace, 671 32 Xanthi, Greece; agaster@pme.duth.gr; 2 School of Medicine, National and Kapodistrian University of Athens, 106 79 Athens, Greece; iseimen@med.uoa.gr; 3 School of Mechanical Engineering, National Technical University of Athens, 157 80 Athens, Greece; dkoulouriotis@mail.ntua.gr

**Keywords:** computer-aided diagnosis, breast cancer, machine learning, deep learning, medical imaging

## Abstract

Breast cancer is the second-leading cause of mortality among women worldwide. However, early detection and diagnosis significantly improve treatment outcomes. In recent years, Computer-Aided Diagnosis (CAD) systems, which leverage Artificial Intelligence (AI) techniques, have emerged as valuable tools for assisting radiologists in the accurate and efficient analysis of medical images. Following the PRISMA guidelines, this study presents the first meta-review that synthesizes evidence from 48 systematic reviews published between 2015 and January 2025. In contrast to previous reviews, which often focus on a single imaging modality or clinical task, our work provides a comprehensive overview of imaging techniques, publicly available datasets, AI methods, and clinical tasks employed in CAD systems for breast cancer diagnosis and treatment. Our analysis shows that mammography is the most frequently applied imaging modality, while DDSM, MIAS, and INBreast are the most commonly used datasets. Among clinical tasks, the detection and classification of breast lesions are the most extensively studied, with deep learning approaches being increasingly prevalent. However, current CAD systems face notable limitations, including the lack of large and diverse datasets, limited transparency and interpretability of AI-based decisions, and restricted clinical integration. By highlighting both the achievements and the limitations, this systematic review aims to support medical professionals and technical researchers in understanding the current state of CAD systems in breast cancer care and to provide guidance for future research directions.

## 1. Introduction

**Figure 1:**
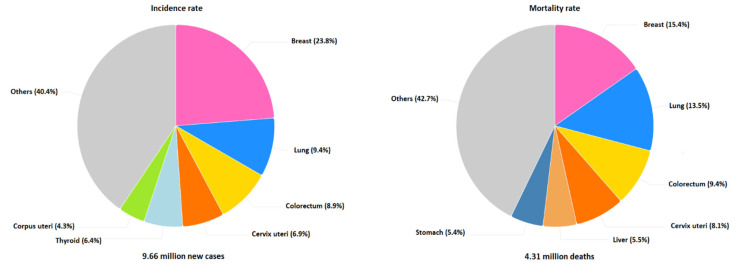
Distribution of cases and deaths for the six most prevalent cancers among women according to Globocan data 2022.

Breast cancer remains one of the most prevalent cancers worldwide, and is one of the leading causes of women’s mortality [1]. It is a genetic disease that arises due to uncontrolled cell growth in the breast tissue, often originating in the ducts or lobules [2]. More women die from breast cancer every year than from any other type of cancer [3]. According to the World Health Organization (WHO) in the year 2022, 2.3 million women had breast cancer, and 666.103 died from this globally. Moreover, it is estimated that 12.4% of women will be diagnosed with breast cancer at some point during their lifetime [4,5]. Figure 1 shows the incidence and mortality rates for the six most common female malignancies in the year 2022. Early detection can reduce the mortality rate, since in the early stage, cancer can respond effectively to treatment and provide useful information about disease development [6,7]. Breast cancer can be effectively diagnosed using several medical imaging examinations, including digital mammograms (DMs), ultrasound (US), Magnetic Resonance Imaging (MRI), Infrared Thermography (IRT), and histological images. Mammography remains the gold standard for breast cancer screening, even though it can yield inconclusive results requiring further assessment.

**Figure 2:**
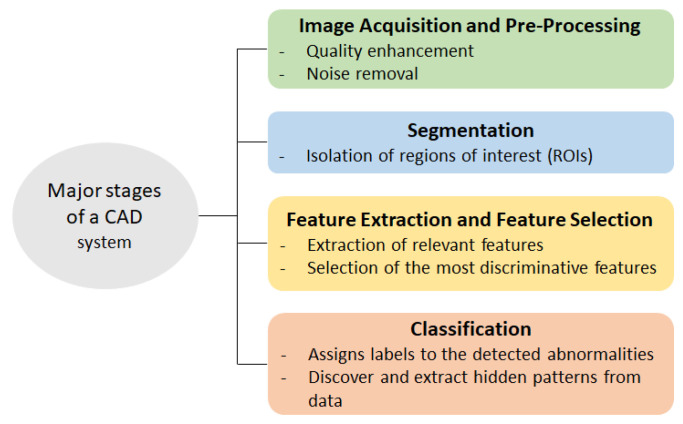
Major stages of a CAD system.

In order to support radiologists in lesion identification, Computer-Aided Diagnosis (CAD) systems with automatic or semi-automatic tools have been developed using Machine Learning (ML) methods. These systems are expected to resolve the operator dependency and reduce the overall medical cost [8,9]. Furthermore, they have the ability to analyze both imaging and/or non-imaging patient data, providing a detailed picture of the patient’s condition [10]. Typically, a CAD system follows a structured pipeline consisting of four main stages, as shown schematically in Figure 2. The first stage is the image acquisition and pre-processing, where the obtained images are enhanced and denoised in order to improve their quality. The second stage is the segmentation, which isolates the regions of interest (ROIs) according to visual characteristics. In the third stage, relevant features such as shape, texture, intensity, and morphological properties are extracted, and the most discriminative ones are selected to minimize classification errors. The group of selected features is used as input to the final classification stage, where an ML technique assigns labels or classes to detected abnormalities and extracts hidden patterns within the dataset, which will be used to predict the future unknown cases [11]. Both traditional ML and Deep Learning (DL) techniques have been extensively used in the medical domain [12,13]. The selection of an appropriate ML technique is critical when developing a CAD system.

The main goal of this systematic review is to provide a comprehensive overview of CAD systems used in breast cancer diagnosis and treatment by examining the application of AI methods based on both traditional ML and DL. This review highlights the associated challenges, limitations, and potential directions for future research. Although several review articles have discussed the application of AI in image-based CAD systems, most of them have focused on a single imaging modality, a limited number of ML algorithms, or a specific clinical task such as detection or classification. Furthermore, these reviews analyze primary studies individually rather than synthesizing findings from multiple systematic reviews, which restricts their ability to capture broader and more accurate methodological trends. To address this gap, the present study performs the first meta-review that synthesizes evidence from 48 systematic reviews published between 2015 and January 2025. It provides an integrated perspective on imaging techniques, publicly available datasets, AI methods, and clinical applications, offering a higher-level understanding of the current progress, limitations, and opportunities in CAD systems for breast cancer care.

The remainder of this paper is structured as follows: In Section 2, the research methodology is presented, explaining the research questions and the criteria for selecting the relevant review articles. In Section 3, we describe the different imaging modalities used for breast cancer diagnosis, and we present the publicly available medical image datasets. Section 4 and Section 5 provide an overview of the most common ML and DL techniques applied in breast cancer CAD systems, along with the specific medical tasks these systems are designed to address. Section 6 explains the performance evaluation metrics used. In Section 7 and Section 8, we discuss the findings of the review, the challenges identified, and some recommendations for future work, followed by the concluding remarks in Section 9.

## 2. Materials and Methods

This systematic review aims to identify various review studies related to breast cancer CAD systems based on medical imaging and ML techniques. The primary aim of this review is to address the following research questions:

1. What is the chronological growth of published reviews on breast cancer CAD systems?
2. Which are the different imaging modalities used for breast cancer diagnosis, and what are their strengths and limitations?
3. Which publicly available medical imaging datasets are utilized by the researchers to develop breast cancer CAD systems?
4. Which are the most common ML techniques currently applied in breast cancer CAD systems based on medical imaging?
5. What performance evaluation metrics are implemented to assess the performance of the developed breast cancer CAD systems?
6. Which medical tasks are most frequently tackled by the breast cancer CAD systems?
7. What are the current challenges and opportunities in the field of breast cancer diagnosis using CAD systems?

The bibliographic literature was thoroughly searched to identify relevant studies. The systematic review followed the recommendations of the Preferred Reporting Items for Systematic Reviews and Meta-Analyses (PRISMA) [14]. However, the protocol has not been registered. For forming our search string, we used three groups of keywords as mentioned in Table 1. The search was performed using the Scopus online digital library, which is a well-established source indexing a wide range of books, journals, and conference proceedings, addressing the research topic of this review. Furthermore, several previous studies and literature reviews, concerning breast cancer CAD systems, have employed Scopus as their primary database [15,16,17]. However, it should be acknowledged that not all databases were searched (e.g., PubMed, IEEE Xplore, and Web of Science), and therefore, some relevant studies may not have been included. Nevertheless, the large number of systematic reviews identified ensures that the findings presented provide a representative and extensive overview of the field.

The data is extracted by applying the search string to the title, keywords, and abstract. Additional filters were also applied for the year, the document type (only review papers were included), and language (English). The time period was set from 2015 to January 2025, within the last decade, and the maximum possible number of publications was investigated for these years. However, some relevant studies may have been skipped unintentionally. After the application of the aforementioned criteria, the search yielded 93 review papers.

**Figure 3:**
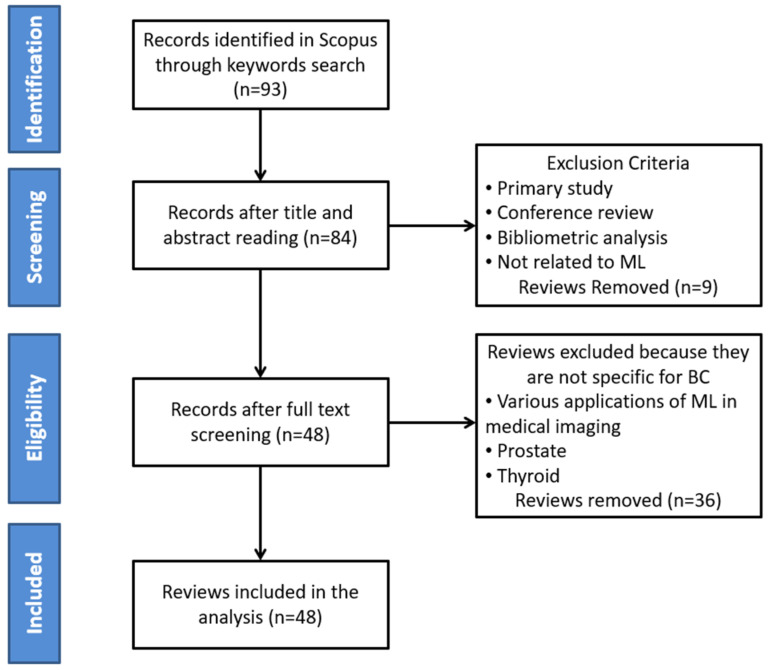
Flow diagram of the record selection process based on the PRISMA approach.

**Figure 4:**
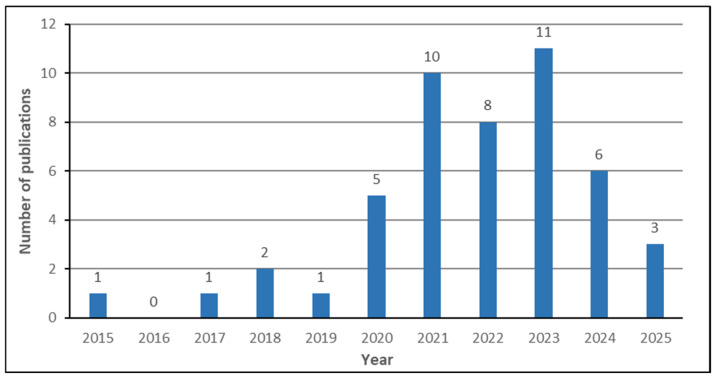
Number of published review papers per year using the specified inclusion criteria.

For narrowing down the initial number of publications, the four-phase flow of the PRISMA approach (labeled “Identification”, “Screening”, “Eligibility”, and “Included”) was used, as depicted in Figure 3. Specifically, all relevant review papers were investigated, but only the ones that satisfied the following inclusion criteria were included: (1) breast cancer was the only disease considered (other diseases were excluded); (2) only complete review articles were considered (primary studies, conference articles/reviews, bibliometric analysis, and abstracts were excluded); (3) at least one ML technique for the development of breast cancer CAD systems was examined; (4) at least one medical imaging modality was used (other diagnosis techniques were excluded); (5) the review was published in between 2015 to January 2025; (6) the performance evaluation metrics of the CAD systems reviewed were recorded; and (7) the review was written in English. From the 93 review papers initially retrieved, only 48 (51.61%) were included in our review. The remaining 45 review papers were excluded from the retrieved list since they did not fulfill the predefined search criteria. Figure 4 presents the number of review papers included in our study based on their year of publication. As can be seen, except for 2016, at least one review was published every year. However, the number of publications increased between 2020 and 2024, with more than five contributions every year. Moreover, the maximum number of contributions was attained in 2023, with 11 contributions. Furthermore, the details of the journal name and the number of reviews being published in each journal are presented in Table 2. From this table, it is shown that 31 journals were used, where only 9 of them, i.e., *Cancers, Computers in Biology and Medicine, Journal of Magnetic Resonance Imaging, Diagnostics, British Journal of Radiology, Expert Systems with Applications, Frontiers in Oncology, Tomography,* and *Computer Methods and Programs in Biomedicine*, had more than one published review.

After selecting the relevant review papers, we proceeded with the extraction of the necessary data related to the purpose of this study. In particular, the information extracted from each review paper, if available, was the image modalities assessed, the available public databases examined, the recorded performance evaluation metrics, the ML techniques presented, the literature databases used, the review period, the role of the developed CAD systems, and the limitations and future recommendations. This process was performed by two researchers in two separate phases. First, both researchers extracted the data separately, and then a cross-validation between researchers was performed to ensure that the data extracted were correct.

## 3. Imaging Modalities and Available Datasets for Breast Cancer Diagnosis

Medical imaging is the most efficient way to assess the presence of breast cancer in its early stages. Several imaging modalities are currently used, including mammography, Digital Breast Tomosynthesis (DBT), ultrasound, Magnetic Resonance Imaging (MRI), histopathology, Thermography, Computed Tomography (CT), Positron Emission Tomography (PET), and Microwave Breast Imaging (MBI) [18,19,20]. Each of these modalities possesses different capabilities. Here, we describe the aforementioned imaging modalities and the publicly available datasets for breast cancer diagnosis.

### 3.1. Mammography

Mammography is the most commonly used and most promising imaging screening technique in breast cancer diagnosis. The main reasons for its extensive use are its fast acquisition, cost-effectiveness, low signal-to-noise ratio, and exploitation facility. Mammography uses low-intensity X-rays in order to detect malignant tumors in the initial stage, before their further development [21,22]. Mammograms can detect breast cancer in women with odd symptoms, as well as evaluate cancer risk in asymptomatic populations. In accordance with the guidelines of the American Cancer Society, women over 45 should undergo annual mammographic screening. Although mammography has several drawbacks, such as the fact that it is an inappropriate screening technique for women with dense breasts or that it has a high rate of false positives, it still remains the gold standard for breast cancer screening and the most reliable and accurate screening technique [23,24,25]. Mammograms can be viewed in various ways in order to obtain more details before diagnosis. The most common views are the craniocaudal (CC) and the mediolateral oblique (MLO), which differ in the capture angle.

Mammograms can be divided into two main categories: Screen Film Mammograms (SFMs) and digital mammograms (DMs). Due to the advancement of technologies, DMs have replaced Film Mammograms as the primary screening modality. Their main advantages are the higher contrast resolution, the ability to enlarge the image layout, and the ability to adjust the image contrast and brightness, helping the radiologists in detecting breast lesions more easily [18]. In the proposed systematic review, mammography was examined in 40 out of 48 reviews included in the study.

### 3.2. Digital Breast Tomosynthesis (DBT)

Digital Breast Tomosynthesis is an advanced imaging technique used for breast cancer screening and diagnosis. This method is similar to mammography, but unlike traditional 2D mammography, which captures a single flat image of the breast, DBT takes multiple X-ray images from different angles to create a detailed, 3D reconstruction of the breast tissue [26,27]. In contrast to conventional mammography, DBT enhances the potential for small tumor detection, especially in women with dense breast tissue. Moreover, by reconstructing 3D images, it minimizes the problem of overlapping tissues that can obscure abnormalities in standard mammograms [28]. The main drawbacks of this method are the greater patient radiation exposure compared to conventional mammography and the additional reading time required by the radiologists due to the number of mammograms obtained. In the present systematic review, 11 reviews assessed the use of DBT in breast cancer CAD systems.

### 3.3. Ultrasound

Breast ultrasound (US) is a non-invasive imaging technique that is widely accessible and extensively used to evaluate breast abnormalities. It is commonly used as an adjunct screening tool, especially for women with dense breast tissue [29,30]. Moreover, it is useful for tumor detection when performing negative mammography [31]. Ultrasound has several advantages over other imaging modalities, including its low cost, fast acquisition, and wide availability. Furthermore, in contrast to X-rays or CT scans, it is suitable for pregnant women and nursing mothers since it is a safe imaging modality that neither involves ionizing radiation nor requires the administration of intravenous contrast agents. Nevertheless, the US has limitations like low specificity, resulting in recalls and unnecessary biopsies for benign lesions; it is highly operator dependent and requires correct probe pressure. Moreover, its capability of discovering contralateral malignant lesions is limited [32,33]. Elastography, 3D ultrasound, and Doppler ultrasound are advanced variations in the traditional US technique. In our systematic review, 35 out of 48 reviews explored the use of the US technique in breast cancer CAD systems.

### 3.4. Magnetic Resonance Imaging (MRI)

Magnetic Resonance Imaging (MRI) is another non-invasive, effective method for early breast cancer detection. Through the use of magnetic fields, it generates high-quality cross-sectional images of the breast, providing accurate three-dimensional (3D) images of the entire breast volume [34]. Therefore, MRI yields greater image clarity and contrast for the assessment of breast cancer compared with other imaging modalities. Moreover, it provides information about the vascularity of the breast tissue and has been proven to be a better screening method in women with dense breasts or at high risk for having breast cancer. This is why the European Society of Breast Imaging (EUSOBI) recommends the use of breast MRI as supplemental screening to women with extremely dense breasts [35]. It has also been shown that occult breast cancers that were not detectable in mammography or ultrasound could be found using MRI [36]. Additionally, it can evaluate the staging period, assisting surgical planning and following up after neoadjuvant chemotherapy (NAC) [37]. Even though MRI exhibits promising advantages, such as high sensitivity and the absence of dangerous ionizing radiation, it may also have some drawbacks; for instance, it is time-consuming, expensive, and has low specificity, which may lead to unnecessary biopsies. Furthermore, it is insufficient in microcalcifications detection, and most of the MRI datasets are not publicly available, limiting its use in research.

Several variations in the traditional MRI method are currently available, such as Diffusion-Weighted MRI (DWI-MRI), Ultrafast Breast MRI (UF-MRI), and dynamic contrast-enhanced MRI (DCE-MRI). In UF-MRI and DCE-MRI, a paramagnetic contrast agent (Gd-DTPA) has to be administered intravenously, which causes images with relatively high intensity in the tumor region, offering an optimal data source for radiomic analyses [38]. In general, these techniques can create much more efficient images with high resolution, for better lesion visualization, improving screening specificity [18,39,40]. In this systematic review, the MRI modality was mentioned in 32 reviews.

### 3.5. Histopathology

Histopathology refers to the microscopic examination of breast tissue. It is a technique applied for many decades, and still remains the benchmark for cancer diagnosis [41]. In order to generate histopathology images, a piece of suspicious breast tissue has to be obtained through either needles or a surgical operation, and then be tested and analyzed by a pathologist. In analysis, the two most commonly staining processes used are Haematoxylin and Eosin (H&E) and Immunohistochemistry (IHC), which produce colored histopathology images for better visualization and in-depth examination of the tissues [42]. Histopathology images are defined as whole-slide images (WSIs) containing multiple ROIs, from which some small patches are extracted, each representing one type of breast lesion only. The development of scanners has digitized histopathological tissue sections and rapidly improved the use of this imaging modality [43]. Currently, histopathological imaging serves as a key tool in cancer diagnosis, as it provides a substantial amount of information essential for medical image analysis. Moreover, they are capable of integrating imaging data with multi-omics features, such as radiomics and genomics, and diagnosing breast cancer with high confidence [44]. However, histopathology images face several limitations. For example, their analysis is a difficult, time-consuming process due to their inconsistent staining. Specifically, their precise analysis requires experienced pathologists who will be able to correctly identify breast cancer based on the color variations in the stained images. Moreover, since biopsies are invasive procedures, considerable time is needed in order to generate digital images from the obtained tissue [45,46]. Only 15 reviews in this systematic review used histopathology images.

### 3.6. Thermography

Thermography is a non-invasive imaging technique used to detect and classify breast cancer based on temperature variations in breast tissue. In particular, sensitive and high-resolution infrared cameras are used to capture temperature maps of the breast. It is based on the principle that malignant tumors exhibit a higher metabolic activity and angiogenesis, leading to localized heat production [47]. Thermography offers a cost-effective, painless, and radiation-free breast cancer screening technique, making it a promising adjunct to other conventional imaging modalities such as mammography, ultrasound, and MRI. Moreover, it is contactless, bringing little discomfort to the patient [48]. Furthermore, in contrast to other imaging modalities, Thermography is very helpful for identifying non-palpable breast cancer [49]. However, challenges such as false positive readings, lack of standardized protocols, and its ability to detect merely surface temperature changes limit its widespread clinical adoption [50]. Thermography was assessed in fourteen reviews included in this systematic review.

### 3.7. Computed Tomography (CT)

Computed Tomography is a widely used imaging modality that provides high-resolution cross-sectional images of the body. Although CT is not a primary tool for breast cancer screening, it is effectively utilized in the staging, metastatic evaluation, and treatment planning for breast cancer patients. It can provide excellent 3D visualization of the breast anatomy, including the location and magnitude of soft tissue lesions, and it can be used as an alternative to 3D MRI [51]. Typically, breast CT has low contrast, and, thus, contrast agents are used in order to enhance image contrast and improve the detection and characterization of breast lesions. Iodine-based contrast agents are most commonly used, injected into a patient’s vein [52]. CT scan is more commonly used than MRI since it is less costly; however, its use for primary breast cancer detection is limited due to lower soft-tissue contrast compared to mammography and MRI [53]. Only nine reviews assessed the use of CT in breast cancer CAD systems.

### 3.8. Positron Emission Tomography (PET)

Positron Emission Tomography, also known as a PET scan, is an advanced radionuclide-based imaging technique used in breast cancer detection, staging, and treatment monitoring [54,55]. PET uses a radioactive substance (radiotracer) in order to visualize and identify changes in metabolic processes and other physiological activities in breast tissue. Typically, the radiotracer is a glucose analog that is injected into a patient’s vein and accumulates in regions with higher metabolic activity, such as cancerous cells [56]. PET can identify and distinguish breast lesions into malignant and benign, based on their glucose uptake. Even though the radiotracer can assist in identifying cancerous tissue, in most cases, PET is not employed independently but is combined with CT or MRI, enhancing its diagnostic accuracy by integrating anatomical and metabolic information [57,58]. PET is particularly valuable for detecting locally advanced breast cancer, cancer metastases, and recurrent disease. However, limitations such as high cost and radiation exposure restrict its use as a primary screening tool for early-stage breast cancer detection. In this systematic review, PET was examined in nine reviews for breast cancer CAD systems.

### 3.9. Microwave Breast Imaging (MBI)

Microwave breast imaging is an innovative, non-invasive imaging technique that utilizes low-power microwave signals in order to detect and characterize breast tumors based on their dielectric properties. It is based on the principle that malignant tissues exhibit distinct electromagnetic characteristics due to increased water content and vascularization, enabling differentiation from healthy parenchyma [59]. A set of specifically designed antennas is used to transmit the microwaves that penetrate the breast tissue and measure their reflection. Based on the information received, an image of the breast tissue can be constructed and analyzed for the detection of abnormalities [60]. MBI has several advantages, including the absence of radiation, cost-effectiveness, painlessness, and the fact that it is not affected by breast density, making it a promising alternative or complementary modality to conventional mammography and MRI. However, further research is needed to establish MBI’s effectiveness since, at present, it is not widely available and is primarily used in clinical trials [61]. The use of MBI in CAD systems was discussed only in three review papers of this systematic review.

Table 3 provides a comparison of the different existing breast imaging modalities along with their best suitability, strengths, and limitations. Mammography and DBT are the primary screening techniques for early breast cancer detection, with the latter offering improved accuracy, especially in patients with dense breasts. Histopathology, on the other hand, remains the gold standard for cancer confirmation, but it is an invasive method that requires tissue samples through biopsy. In terms of staging and metastasis evaluation, it is observed that MRI, CT, and PET are recognized to be among the advanced imaging modalities used. MRI, especially, can be used for the initial staging of breast cancer due to its capability for soft tissue imaging and lesion interpretation [62]. However, it remains costly and its assessment requires skilled radiologists. Thermography and MBI are painless and radiation-free potential alternative screening techniques for early breast cancer detection, but they need additional clinical validation.

**Figure 5:**
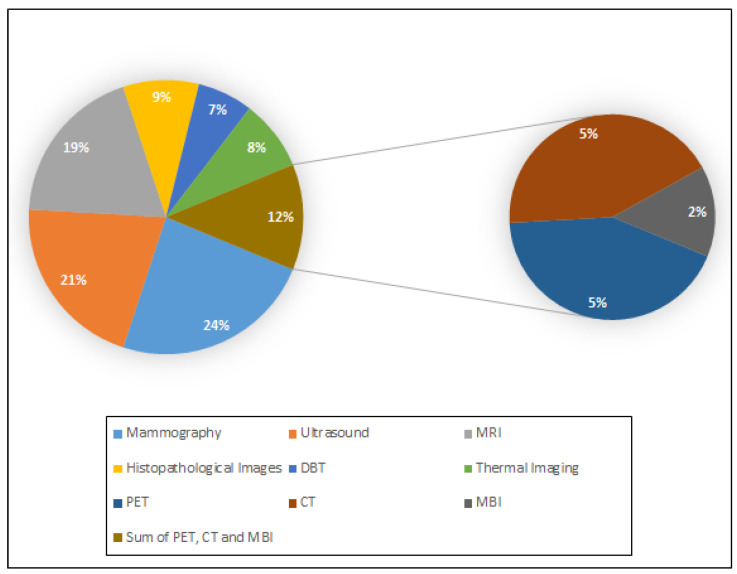
Pie chart of different imaging modalities used in breast cancer CAD systems.

From the data extraction process, Figure 5 shows a pie chart of the used imaging modalities in the included systematic reviews. Among 48 systematic reviews, 31 (64.6%) assessed the use of more than one imaging modality in breast cancer CAD systems. Each pie sector shows the extent of use of each of these different modalities in percentage form. As it is shown, mammography is the most commonly used imaging modality in different CAD models for breast cancer diagnosis. This is because the existing publicly available image datasets have a limited range of data. Specifically, the digital mammography datasets are typically large, but only limited data are included in US, MRI, PET, and CT datasets [42]. Furthermore, MRI datasets with labeled tumor images are scarce, making it difficult to train ML models effectively [63].

**Figure 6:**
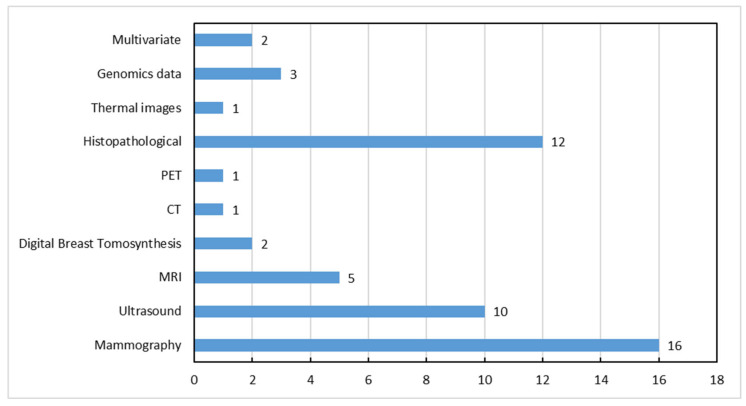
Dataset distribution among the different imaging modalities.

Even though the majority of studies relied on private datasets, a total of 49 publicly available datasets have been reported in the included reviews, with three of them including more than one modality, bringing the total number of datasets to 53. However, some datasets are not completely open-access, requiring approval or a license agreement to access them. Their distribution among the different imaging modalities is shown in Figure 6, from which it can be confirmed that mammography public datasets are more abundant compared to other modalities. Table S1 in the Supplementary Materials provides the most popular datasets used for breast cancer diagnosis using CAD systems, as well as important details for each one, such as the number of images, the image format, and their accessible link. Furthermore, the acronyms of these datasets are given in Table S2 in the Supplementary Materials.

The most commonly utilized dataset was the DDSM dataset, which was reported in 31 reviews (64.58%), followed by the MIAS and the INBreast datasets, which were both reported in 29 reviews (60.42%). Another popular dataset was the BreaKHis dataset, which was reported in 11 reviews (22.92%), as well as the Wisconsin breast cancer datasets, which were reported in 12 reviews (25%). These datasets are the most popular, probably because they not only include a large set of images or clinical data but also because they are completely open-access, without the need for registration or approval.

## 4. Machine Learning Techniques Applied in Breast Cancer CAD Systems

**Figure 7:**
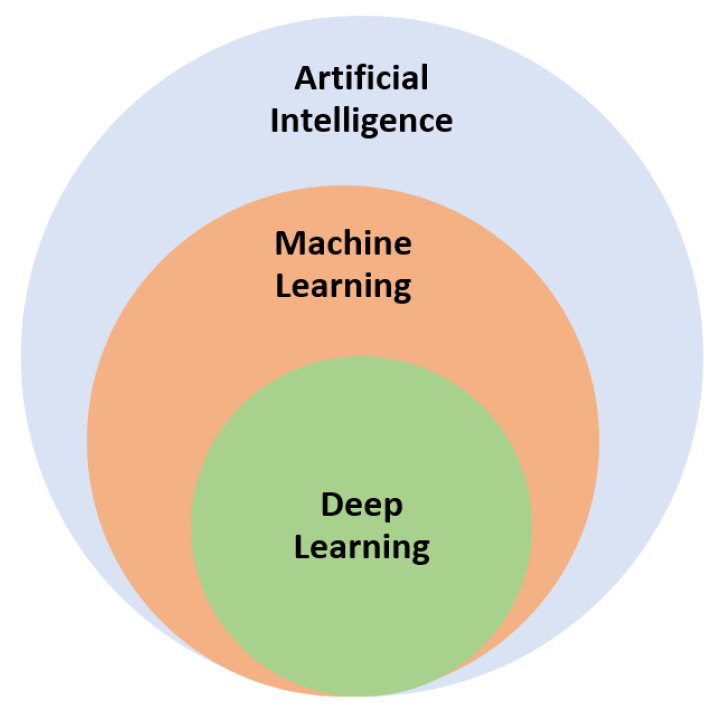
Relationship between Artificial Intelligence, Machine Learning, and Deep Learning.

Machine Learning is a term used to describe a group of techniques that can identify patterns in data and use them to make predictions or decisions without explicit programming. Specifically, ML is a subset of AI, and its methods can learn directly from data, eliminating the need for numerous expert rules or precisely representing every environmental element [64]. ML techniques have been widely used in breast cancer research to assist physicians by providing a second opinion and therefore reducing potential human errors. With their use, decision support systems have been implemented to achieve high accuracy and respond immediately in emergency cases, helping physicians in their daily activities. However, traditional ML approaches require handcrafted features derived from raw image data as pre-processing steps, which is a laborious and time-consuming process. To overcome this limitation, Deep Learning approaches have been introduced. DL is a specialized branch of ML that utilizes artificial neural networks with multiple layers (deep neural networks) to process complex data [65]. They can process raw images and extract the appropriate data by mathematical optimization and multiple-level abstractions, and effectively differentiate them into classes. However, DL models require a large amount of high-quality training data as well as substantial computational resources for effective training [66]. Although both approaches have shown promising results in breast cancer diagnosis, recently, DL approaches have been primarily utilized in breast cancer CAD systems due to their exceptional accuracy and optimal performance. Figure 7 presents the relationship between AI, ML, and DL.

Machine learning techniques are broadly categorized into supervised and unsupervised learning based on the nature of the data and the learning process. Supervised learning relies on labeled datasets, where each input is mapped to a pre-defined output, and the algorithm learns based on these input-output pairs in order to make predictions or classifications. In contrast, unsupervised learning deals primarily with unlabelled data, identifying trends, patterns, structures, and undetected knowledge without predefined outputs [67].

**Figure 8:**
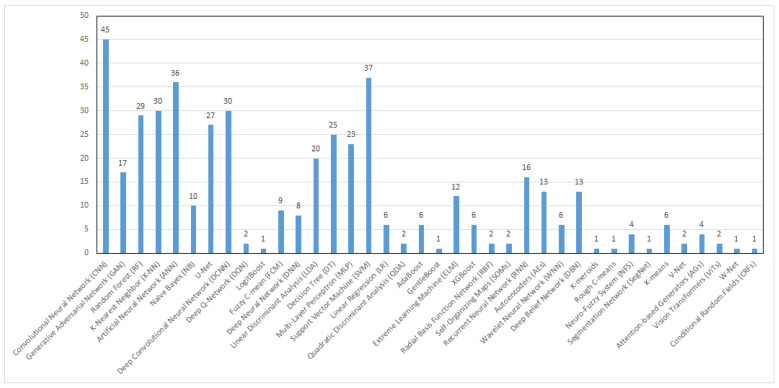
Extent of use of each Machine Learning technique.

Several ML techniques used in the development of breast cancer CAD systems were mentioned in the review papers included in this systematic review. The extracted details are presented in Table S3 [11,15,16,17,18,19,20,39,40,42,43,60,61,65,66,68,69,70,71,72,73,74,75,76,77,78,79,80,81,82,83,84,85,86,87,88,89,90,91,92,93,94,95,96,97,98,99,100] in the Supplementary Materials. Since more than one technique was mentioned in each review, all techniques are recorded in the corresponding cell of the table. Furthermore, Figure 8 illustrates the extent of use of each recorded technique, and a brief description of the most commonly used ML techniques is provided. However, it should be noted that the reviewed systematic reviews did not consistently report the specific hyperparameter tuning strategies applied in the primary studies and, therefore, such methodological details were considered beyond the scope of this meta-review.

### 4.1. Artificial Neural Network (ANN)

**Figure 9:**
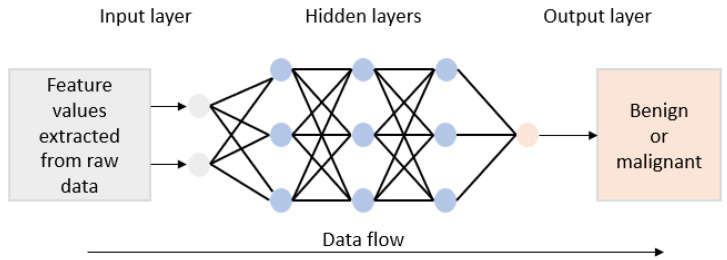
Architecture of an ANN for breast cancer diagnosis having multiple hidden layers.

Artificial neural networks are computational models inspired by the human brain’s mechanisms for processing information. They consist of interconnected layers of neurons that process and transmit data through weighted connections and activation functions, such as ReLU (Rectified Linear Unit), sigmoid, and tanh, enabling the learning of complex patterns [15,39]. Typically, an ANN includes an input layer for the raw data acquisition, several hidden layers for data transformation and feature extraction, and an output layer that provides the network’s final output [19,65]. The network’s learning process involves adjusting the connection weights using optimization techniques like backpropagation and gradient descent to minimize the error between predicted and target outputs [85,97]. Owing to their ability to model complex, non-linear relationships, ANNs are widely applied in biomedical engineering and medical diagnosis, where they can adapt to varying dataset sizes and handle incomplete or missing data effectively [101]. In this systematic review, ANNs were considered in 36 review papers, which corresponds to 75% of the included reviews. Furthermore, specific types of ANNs are also considered, such as the Multilayer Perceptron (MLP), which is one of the most commonly used ANN architectures [72,75,85,91,92,93,94,95], and Wavelet Neural Networks (WNNs), which are an extension of traditional ANNs and integrate wavelet transformations by replacing traditional activation functions with wavelet basis functions [11,16,60,74,79,96]. Moreover, 12 reviews examined the use of Extreme Learning Machines (ELMs), which are specialized types of ANN designed to improve training speed and efficiency by assigning random values to the weights and biases of the hidden layers [16,17,19,39,40,60,71,72,75,80,82,91]. The architecture of an ANN for breast cancer diagnosis having multiple hidden layers is given in Figure 9.

### 4.2. Convolutional Neural Network (CNN)

Convolutional neural networks are DL frameworks designed for processing structured grid data, such as images, and have revolutionized medical diagnostics, particularly for breast cancer detection and classification [16,39,102]. Their success stems from an architecture capable of identifying both local and global features of the input data. A CNN consists of convolutional, pooling, and fully connected layers, each performing a different task [68,76]. The convolutional layers act as feature extractors. With the use of filters (kernels), they scan through the image and detect edges, textures, and patterns. By stacking multiple convolutional layers, CNNs can progressively learn more complex features and become more effective [103,104]. The pooling layers reduce the spatial dimensionality and the network parameters, while the fully connected layer utilizes the extracted features to classify the input image into predefined classes [87,89]. The CNNs are categorized as De novo CNNs, which are CNNs trained from scratch, and Transfer Learning-based CNNs, which are pre-trained networks that are fine tuned and adjusted to solve specific tasks [84]. Furthermore, they can be categorized into shallow, moderately deep, and deep, according to the number of layers used [81,85]. Deep CNNs typically include dozens or even hundreds of convolutional layers, making them highly effective for handling large-scale image datasets [87]. The classification of CNNs based on the number of layers used is provided in Table 4.

As opposed to other methods, CNNs require less pre-processing, can combine feature extraction and image classification, and are resistant to image noise and local geometric distortions. However, they require a large number of images for training, and it is difficult to achieve good performance when using limited datasets, even though transfer learning has been shown to be an effective approach for dealing with small datasets. Several well-known CNN architectures for breast cancer diagnosis have been used in the studies mentioned in the included reviews, such as AlexNet [68,73,76,77,78,83,92], VGGNet [68,69,70,71,72,76,81,82,83,84,90,92,98], GoogLeNet [70,73,88,90,98], ResNet [72,76,86,87,88,89,90,99,100], DenseNet [71,73,76,81,84,92], EfficientNet [71,78,83], Yolo [68,76,78,89,92], and Faster R-CNN [72,73,84,90,99,100], achieving high performances. Specifically, according to Jannatdoust et al. [100], the U-Nets, which are a specialized category of CNNs designed for semantic segmentation using an encoder–decoder architecture with skip connections, have achieved benchmark accuracies of up to 99.5% and demonstrated robust performance in tasks such as lesions segmentation. Moreover, Mahoro et al. [83] examined the use of CNNs in different imaging modalities, achieving high accuracy, which ranged between 98.33% and 99.33%.

### 4.3. Support Vector Machine (SVM)

Support Vector Machine is a widely used Machine Learning approach in biomedical imaging, particularly in breast cancer diagnosis. It is a supervised classifier originally introduced by Vapnik in 1999 [105]. By mapping the labeled input data into a high-dimensional feature space, SVMs can effectively separate them into classes (e.g., benign vs. malignant lesions), using an optimal hyperplane as a boundary that maximizes the separation margin between them, ensuring that the model generalizes well to new data samples. The hyperplane is fully specified, and the training samples that lie closest to it represent the support vectors [65,106,107]. The SVM approach, unlike other conventional classification methods that minimize empirical risk or maximize the output based on the training set, minimizes structural risk, i.e., the probability that new data samples will be classified correctly for a fixed data probability distribution [86]. SVM models can be efficiently employed either for linear or non-linear separable datasets. Specifically, the “kernel trick” allows SVMs to classify non-linearly separable data by transforming it into a space where linear separation is possible. Depending on the chosen kernel function and scale, SVMs can be further divided into linear, quadratic, fine-Gaussian, medium-Gaussian, and coarse-Gaussian variants [71,108]. Among them, the RBF kernel is the most commonly applied in medical imaging tasks because it can handle non-linear feature relationships while avoiding the oversimplification of the decision boundary, such as linear kernels, as well as the overfitting risks of polynomial kernels.

One of the key strengths of SVMs in breast cancer diagnosis is their ability to work with different imaging modalities and integrate multiple extracted features, such as texture, shape, and intensity features. Furthermore, their ability to handle high-dimensional feature spaces makes them particularly useful when dealing with the complex and subtle variations present in medical images. Moreover, they are highly robust techniques that perform well with unstructured or semi-structured data and can tackle overfitting issues [15,109,110]. However, they are sensitive to the selection of the kernel function and its parameters, particularly when dealing with large datasets [111]. In addition to that, they require well-curated and high-quality labeled data for training. In the presented study, SVM classifiers were examined in 37 reviews, which represent 77.1% of the total considered studies. As it was observed, classifying breast cancer using SVM classifiers can accomplish excellent accuracy values. In particular, the highest accuracy achieved was 100%, as stated by Yassin et al. [19], while Liew et al. [43] reported accuracy values that ranged from 97.13% to 99%. Excellent performance was also achieved in the work of Sharma et al. [112], where a CAD system for the classification of malignant and non-malignant mammogram patches was developed. Zernike moments of different orders were computed for all the extracted patches and were fed into an SVM classifier. The system attained 99% sensitivity and 99% specificity when the IRMA dataset was used, and 96% sensitivity and 96% specificity when the DDSM database was utilized.

### 4.4. K-Nearest Neighbor (K-NN)

K-Nearest Neighbor is a simple, non-parametric, supervised learning classifier proposed by Friedman et al. in 1977 [113], that has been widely used for pattern recognition and classification tasks [65,114]. It classifies a new input sample based on the majority class of its k nearest neighbors in the training dataset, using distance metrics such as Euclidean, Manhattan, or Mahalanobis distance, with Euclidean being the most commonly used [97,114,115,116]. K-NN’s non-parametric nature makes it highly adaptable to different types of datasets, particularly in breast cancer diagnosis, where tumors exhibit a wide range of morphological characteristics [43,114]. Furthermore, it is a faster method compared to other frameworks that require training, and it can be interpreted easily. However, for large datasets, K-NN can be slow and memory intensive due to distance computations. Its performance is also sensitive to dataset imbalance, feature scaling, and the choice of k, as small k values increase susceptibility to noise, while large k values may oversimplify classification [86,117,118].

In the proposed systematic review, 30 out of 48 reviews explored the use of K-NN classifier in breast cancer CAD systems. Rajaguru et al. [114] showed that the K-NN method outperformed the Decision Tree method in breast cancer classification. Raghavendra et al. [119] classified 690 digitized mammogram images from the DDSM database. The classification was performed in three classes, i.e., normal, benign, and malignant, where each class had the same number of mammograms. They used nine different classifiers, with K-NN achieving the highest average accuracy at 98.69%. Murtaza et al. [120] developed a classification model in order to classify breast cancer into eight distinct subtypes. For their experiments, they used the BreakHis publicly available dataset. They analyzed six different machine-learning classifiers, where K-NN had the best performance and obtained the highest accuracies.

### 4.5. Decision Tree (DT) and Random Forest (FR)

The Decision Tree is an efficient, non-parametric, supervised learning classifier commonly used for breast cancer diagnosis. Based on a “divide and conquer” approach, it recursively splits the dataset according to specific features during the training process, building the model in a tree format. In general, a DT classifier can be expressed as a set of decision rules that have been organized in a tree structure [97,121]. A typical tree consists of a root node that represents the entire dataset, internal/decision nodes that apply attribute-based test conditions, and terminal (leaf) nodes that correspond to class labels or predictions. For every new input sample, the tree is traversed from root to leaf to determine the predicted class [122,123]. DTs can be categorized as coarse, medium, and fine based on their depth and granularity of splits, supporting 4, 10, and 100 approximate splits, respectively. One of the major advantages of DTs in medical applications is their interpretability, since the physicians can easily follow the path of the tree to understand the logic behind a classification decision, making it easier to trust. Furthermore, they are computationally efficient without the need for data normalization and can handle multi-classification problems. On the other hand, a major limitation is their tendency to overfit the training data, meaning they may not generalize well to unseen patient samples, which can lead to misdiagnosis [15,85,124]. An improvement of DT is the Random Forest classifier. It is an ensemble learning method that creates multiple DTs during the training stage, using random subsets of patient data and features. When a new input appears, each tree performs its classification independently, and the final classification is determined by a majority vote from all trees [88,125]. This way, the accuracy is enhanced. Additionally, by randomly selecting a subset of features for each tree, the model reduces the risk of feature bias, ensuring a more balanced and generalizable prediction. However, an RF model can be computationally expensive, especially when it contains a large number of trees. Moreover, when data is extremely unbalanced, the model gives suboptimal results [126,127].

The use of RF in breast cancer CAD systems was assessed in 26 review papers included in the current systematic review, while DT was assessed in 25 reviews. Several studies achieved high accuracy values, such as Azar et al. [128], who presented a decision support tool for the detection of breast cancer based on three types of DT classifiers: single Decision Tree (SDT), boosted Decision Tree (BDT), and Decision Tree forest (DTF). According to their results, the DTF outperformed the other two classifiers, achieving the best accuracy during validation at 97.51%. Mohanty et al. [129] proposed a CAD system for cancer detection from DMs. They employed Gray-Level Co-occurrence Matrix (GLCM) and Gray-Level Run-Length Matrix (GLRM) features to distinguish benign–malignant masses from ROI patches using a DT classifier. The maximum accuracy achieved was 97.6% when 19 features were used. The accuracy was decreased to 93.6% when only 12 of the 19 features were used. Furthermore, the proposed system achieved an AUC of 99.5%. Asri et al. [130] compared the performance of four different ML classifiers using the Wisconsin Breast Cancer (original) datasets. They applied the C4.5 algorithm, which is the most widely used DT algorithm, obtaining 95.13% accuracy. Ribeiro et al. [131] introduced a CAD system for the classification of mammography images, aiming to identify the presence of breast masses. They used the unsupervised Optimum-Path Forest (OPF) classifier, and compared it against two other unsupervised techniques, Gaussian Mixture Model (GMM) and k-Means, using texture features. According to the results, the OPF classifier obtained the best average accuracy of 99.9%. Ghongade et al. [132] implemented a CAD system for the classification of breast tumors in digital mammograms. Initially, GLCM features were extracted from ROIs, followed by a fast correlation-based feature selection technique. The derived features were classified by an RF with an accuracy of 97.32%. Gubern-Merida et al. [133] developed an automated breast cancer localization CAD system for DCE-MRI. In the final stage of the area classification, five different classifiers were compared, i.e., LDA, K-NN, Gentleboost, SVM, and RFs. The best results were obtained using RFs with an average sensitivity of 89% at four false positives per normal case (91% and 86% for mass-like and non-mass-like malignant lesions, respectively).

### 4.6. Discriminant Analysis (DA)

Discriminant Analysis is an advanced statistical supervised-learning classifier used for classifying observations into predefined classes based on predictor variables. It employs adequate dimension reduction from the initial data space to discriminate between the required number of classes. Specifically, it constructs a discriminant subspace that enhances class separability by minimizing within-class scatter and maximizing between-class scatter [97,134,135]. This technique is easy to apply and particularly valuable in cases where the number of features exceeds the number of observations, such as in radiomics, where numerous textural, shape, and intensity features are extracted from medical images in order to enhance classification [136].

In the included reviews of this systematic review, two different DA methods were examined, the Linear Discriminant Analysis (LDA) and the Quadratic Discriminant Analysis (QDA). In particular, LDA was examined in 20 reviews and QDA in 2 reviews. The difference between them is that LDA assumes that different classes have the same covariance matrix, while QDA allows each class to have its own covariance matrix. Furthermore, LDA finds a linear decision boundary for the class separation, while QDA provides a quadratic decision boundary [71,134]. In the work of Li [137], a framework for breast tissue classification was proposed using a modified Fisher’s discriminant analysis algorithm. It utilized mammograms from the mini-MIAS database, achieving an accuracy of 94.46%. Esener et al. [138] designed and validated an automatic CAD system for breast cancer diagnosis using mammograms obtained from the IRMA dataset. According to their results, SVM and Fisher’s LDA had similar performance with maximum recognition accuracy at 94.67% and 94.17%, respectively. QDA was used in the work of Spanhol et al. [139] who presented a pattern recognition system for the discrimination of histopathology images obtained from the BreaKHis database. The QDA model achieved an average accuracy of 84.2%.

### 4.7. Generative Adversarial Networks (GANs)

Generative Adversarial Networks are DL models designed to generate synthetic data through a unique adversarial framework comprising two competing sub-models: the generator and the discriminator [140]. The generator produces synthetic samples that mimic the distribution of real data, while the discriminator attempts to differentiate genuine from fake samples. During training, both networks iteratively refine their parameters until an equilibrium is reached, at which point the discriminator achieves near-optimal accuracy [141]. GAN models fall under unsupervised learning and have been widely used in medical imaging for data augmentation, image reconstruction, and segmentation. Due to their ability to generate synthetic training data, they can be effectively employed in cases where it is difficult to collect a large number of images needed to train a classifier, such as infrared thermal images, MRI, and PET scans, augmenting the existing datasets without compromising data privacy regulations [141,142]. Furthermore, GAN-based architectures can help in anomaly detection by learning the normal structure of breast tissue and identifying abnormalities that deviate from typical patterns. However, the primary limitation of GANs is their training instability and high computational demand, often requiring extended training time [143]. In addition, the increasing use of GANs for data augmentation raises concerns about distributional bias, as synthetic images may not fully represent real-world variability or could introduce artifacts that reduce the model’s generalizability. Recent studies attempt to mitigate bias by combining GANs with traditional augmentation methods, employing conditional GANs in order to generate more realistic and diverse images, or using GANs as a complement to real patient data rather than as a replacement. Thus, even though GAN-based augmentation is a powerful tool for advancing CAD systems, its use should be carefully validated before clinical application.

In this systematic review, GANs were considered in 17 review papers, which correspond to 35,47% of the included reviews. Guan et al. [144] used both affine transformations and a GAN model to augment the training data of a CNN classifier. The original images were obtained from the DDSM database. According to their results, the addition of GAN ROIs to the training data can improve classification performance, and the improvement is about 3.6% better than adding affine transformation ROIs. Fan et al. [145] employed a GAN model to produce super-resolution apparent diffusion coefficient (ADC) images and assess their clinical utility by performing a radiomics analysis for predicting the histologic grade and Ki-67 expression status of breast cancer. In the work of Swiecicki et al. [146], a GAN model was presented for the detection of anomalies in DBT images. The detection system showed promising results, as it was able to identify abnormal locations even though it was trained using normal-only images. Singh et al. [147] proposed a conditional GAN (cGAN) for breast tumor segmentation within an ROI already extracted from a mammogram. The generative network learns to detect the tumor area and generates a binary mask that outlines it. On the other hand, the adversarial network learns to distinguish between real and synthesized segmentations. As a result, the generative network is enforced to generate binary masks that are as realistic as possible. The created binary masks are subsequently classified by a CNN-based shape discriminator. The proposed segmentation model obtained a high Dice coefficient and Intersection over Union (IoU) of 94.07% and 87.03%, respectively.

## 5. Medical Tasks Tackled by Breast Cancer CAD Systems

**Figure 10:**
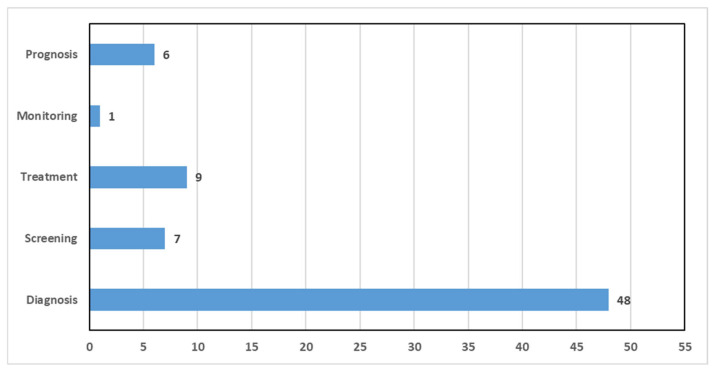
Medical tasks investigated by the selected reviews.

CAD systems aid in automating various medical tasks related to breast cancer analysis. Several tasks have been examined in the reviews included in this systematic review, which can be grouped into five main themes based on their primary focus area. This thematic grouping was intended in order to highlight the use of CAD applications across different stages of breast cancer care. Figure 10 presents the distribution of the selected reviews for these medical tasks. As shown, the diagnosis tasks such as breast cancer classification, mitosis detection, and prediction of breast cancer molecular subtype, were investigated in all the reviews. Treatment and screening tasks followed, with nine reviews (18.75% of the selected reviews) and seven reviews (14.58% of the selected reviews), respectively. Prognosis was investigated in six selected reviews, while monitoring was only in one review. Furthermore, we found that the majority of reviews examined CAD implementations related to only one task, nine reviews investigated three tasks, while only one review investigated all five tasks.

## 6. Evaluation Metrics

The evaluation of a CAD system for breast cancer diagnosis is an important step that can demonstrate its effectiveness. During this process, we can understand the system’s performance quantitatively as well as identify and resolve any underlying problems. There are various evaluation metrics, such as the confusion matrix, the receiver operating characteristic (ROC) curve, the area under the ROC curve (AUC), and so on. The most repeated metrics clearly mentioned in the included reviews are as follows: accuracy, sensitivity, specificity, precision, F1-score, Dice Similarity Coefficient (DSC), and AUC. They are mostly calculated depending on the following parameters [39,43,82]:

- True positive (TP), i.e., the number of patients for whom the system predicts that they are suffering from cancer, and they actually do.
- True negative (TN), i.e., the number of patients for whom the system predicts that they are not suffering from cancer, and they actually do not.
- False positive (FP), i.e., the number of patients who are predicted to be suffering from cancer but are not, in fact, suffering from cancer.
- False negative (FN), i.e., the number of patients predicted as healthy patients but in fact, they are suffering from cancer.

Accuracy (Acc): The accuracy score is determined by dividing the correct predictions made by the total number of predictions. It represents how near the predicted class is to the actual one. The accuracy can be defined using Equation (1).

(1) $$Accuracy=\frac{TP+TN}{TP+FP+TN+FN}\;$$

Sensitivity (Sn): This is determined by dividing the number of abnormal cases that are predicted correctly by the total number of abnormal cases. It can be computed using Equation (2).

(2) $$Sensitivity=\frac{TP}{TP+FN}$$

Specificity (Sp): This is the proportion of the normal cases that are correctly identified. It is calculated according to Equation (3).

(3) $$Specificity=\frac{TN}{TN+FP}$$

Precision (Pr): Precision is calculated as the ratio of the correctly predicted abnormal cases to the total number of cases that were predicted as abnormal. In medical image diagnosis, both sensitivity and precision should be high in order to avoid the misdiagnosis of patients. Precision can be expressed using Equation (4).

(4) $$Precision=\frac{TP}{TP+FP}$$

F1-score: It is a weighted harmonic mean of sensitivity and precision and determines the system’s accuracy in each class. It is a metric usually used when the dataset is imbalanced. It can be defined by Equation (5).

(5) $$F1-score=2\times \;\frac{\left(Precision\;\times \;Sensitivity\right)}{\left(Precision+Sensitivity\right)}$$

Dice Similarity Coefficient (DSC): It is a statistical tool that shows the overlap between what the system predicts as abnormal and what is actually abnormal. In breast cancer CAD systems, DSC is commonly used to evaluate the accuracy of image segmentation. It focuses only on the positive/abnormal class, which is especially useful in medical image segmentation. It can be calculated according to Equation (6).

(6) $$DSC=\frac{2\;\times \;TP}{(2\;\times \;TP)+FP+FN}$$

AUC: It is a common metric that represents a way to choose optimal systems and ignore sub-optimal ones. Specifically, it measures how well the system separates malignant from benign cases. It is the area under the ROC curve, which is a two-dimensional graph of the true positive rate (sensitivity) versus the false positive rate (1-specificity) within different thresholds. The AUC takes a value between 0 and 1, where values close to 1 indicate reliable performance of the system.

## 7. Discussion

Breast cancer is one of the most prevalent and life-threatening cancers affecting women worldwide. According to the WHO, it is the most commonly diagnosed cancer globally. Therefore, the early detection of breast cancer is essential for the reduction in the mortality rate, especially in high-risk cases. To support radiologists in the difficult task of breast cancer diagnosis, CAD systems with automatic or semi-automatic tools have been developed that combine ML techniques with advanced imaging modalities to enhance lesion detection, classification, and overall diagnostic accuracy. In fact, a common finding among the studies included in this systematic review is that CAD systems, when used as a second reader, significantly improve radiologist performance in the diagnosis of breast abnormalities, without a proportional increase in false positives [11,72,74,87].

Our review comprehensively described and compared the different existing breast imaging modalities, as well as the publicly available datasets most commonly used by the researchers, since the already performed surveys are restricted in terms of imaging approaches. Specifically, most of the surveys describe only a few breast imaging approaches, and in several cases, only one modality is assessed [69,70,71,72,80,81,82,93]. Seeing the single modalities, MRI has the highest sensitivity regardless of breast type, density, and patient history. On the other hand, mammography is less sensitive, especially in dense breasts, but the advancement of machine learning and deep learning approaches in DBT, whose use is currently limited, is expected to revolutionize the detection and treatment of breast cancer. Studies utilizing mammography remain the most prevalent, owing to the fact that most publicly available datasets are related to this modality, as shown in Figure 6. Moreover, only a limited number of datasets are available related to novel and ancillary techniques such as Thermography, PET, and MBI. The DDSM, the MIAS, and the INBreast are the most popular datasets and have been pivotal in benchmarking CAD performance. Nevertheless, the databases present varying trade-offs in terms of data quality and scale. For instance, the DDSM provides a large-scale dataset with extensive annotations, making it suitable for robust model development. In contrast, the MIAS dataset offers a relatively small number of low-resolution images and is affected by considerable noise. On the other hand, the INbreast dataset contains high-resolution, high-quality images, but with a comparatively small dataset size [148]. It should also be noted that most of the available breast cancer datasets are carefully processed, refined, and close to balanced. However, imbalanced datasets may lead to inaccurate diagnoses, whose consequences may be severe.

**Figure 11:**
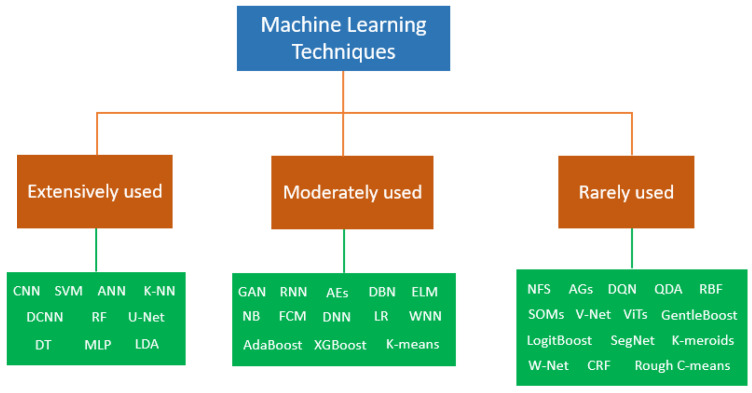
Machine Learning techniques categorized according to their frequency of use in breast cancer CAD systems.

This review examines a wide range of ML techniques employed in CAD systems. As illustrated in Figure 8, the application patterns of these techniques vary considerably, with some being applied extensively, others moderately, and a few only rarely. Figure 11 presents the categorization of ML techniques according to their frequency of use in breast cancer CAD systems. Based on our review, SVM and CNN are the most accurate classifiers, with reported accuracies reaching up to 100% in certain studies [19,43,83,112]. The most commonly used CNN architectures are AlexNet, VGGNet, GoogLeNet, ResNet, DenseNet, EfficientNet, U-Net, Yolo, and Faster R-CNN [68,73,76,84,90,92]. Additionally, the majority of the primary studies in the included reviews used supervised learning techniques, while unsupervised learning techniques were mainly used in segmentation tasks. Furthermore, most of the included reviews examined the use of single classification techniques, even though ensemble methods provide higher accuracy than the single approaches. Specifically, only one review focused on the use of ensemble classification methods in breast cancer diagnosis [95]. Likewise, only one review assessed the detection and classification of breast abnormalities using temporally sequential mammograms, owing to the limited availability of open-access datasets [69]. Another significant observation is that most of the researchers have developed CAD systems aimed at binary classification of lesions, differentiating between malignant and benign, rather than adopting a more comprehensive three-class classification: normal tissue, malignant, and benign. Therefore, there is an ongoing need for the development of CAD systems capable of distinguishing ROIs into three distinct classes.

Furthermore, we assessed the medical tasks that are being tackled by breast cancer CAD systems. Although this is an important task that can determine which medical tasks are well-covered, as well as reveal research gaps, it has been evaluated in a limited number of reviews [95,99]. According to the results presented in Section 5, the diagnosis task was most frequently addressed in the included reviews. In fact, this is not surprising, as early diagnosis of breast cancer can influence the treatment of the disease and reduce the mortality rate. Additionally, early detection has the potential to eliminate the necessity for numerous diagnostic procedures and significantly reduce healthcare costs by prioritizing cost-effective early-stage interventions over more expensive late-stage treatments. Therefore, the majority of researchers were interested in designing accurate CAD systems that could assist physicians in accurately detecting both malignant and begin lesions. Moreover, their interest was affected by the publicly available datasets, which mainly concern the diagnosis task, while only a few of them concern the remaining tasks.

## 8. Challenges and Future Work

Despite the positive results presented in the reviewed literature and the considerable progress achieved in the medical field over the last decade, with the application of machine and deep learning techniques to breast cancer CAD systems, several critical limitations and challenges persist that prevent the successful integration of CAD systems in clinical practice.

The primary challenge observed in the reviewed studies was the insufficient availability of large-scale, high-quality, and publicly annotated datasets. ML and especially DL techniques require huge training data because, to a large extent, their performance depends on the size and quality of the dataset. However, the creation of an adequate medical imaging dataset is difficult, since the annotation of medical images is tedious, time-consuming, expensive, and requires considerable effort from several experts to eliminate human error. The collection of data is also affected by the number of patients examined, as well as privacy regulations and institutional data protection policies in various healthcare centers. Consequently, mammography datasets are typically more extensive, containing data from thousands of patients, whereas MRI, PET, and CT datasets tend to include information from a much smaller number of patients. As a result, fewer studies have been conducted focusing on these imaging modalities. Another critical challenge is the limited interpretability of DL CAD systems. While these models often achieve high accuracy, in many cases, their decision-making process is not transparent due to the “black box” nature of DL algorithms. This opacity is particularly problematic in clinical settings, as it is essential for medical experts to know and understand the rationale behind diagnostic decisions for both ethical and practical reasons. The inability of DL models to provide clinically interpretable justifications for predictions makes it difficult for clinicians to trust and adopt their results into diagnostic workflows.

Furthermore, the lack of standardization across the implemented studies is affecting the comparability and reproducibility of the developed CAD systems. Specifically, the majority of studies relied on private datasets, using different pre-processing techniques, model architectures, validation strategies, and performance metrics, making it difficult to compare, establish benchmarks, or draw generalizable conclusions. As a consequence, the applicability and reliability of the developed CAD systems in real-world clinical environments remain uncertain. Moreover, another significant barrier indicated by several studies is the computational requirements associated with developing and training DL models for breast cancer imaging. These models often require access to high-performance computing resources, particularly when working with high-resolution medical images, such as mammograms, MRI, or PET scans. Additionally, the manual effort involved in annotating training data further increases the development burden. Access to such resources can be costly and may limit the scalability of CAD systems, highlighting the importance of developing more efficient and cost-effective models suitable for real-world clinical integration.

In addition to technical challenges, several practical and ethical barriers hinder the real-world implementation of CAD systems for breast cancer diagnosis. Specifically, most of the developed systems remain at an experimental or research stage, with only a limited number having received approval from regulatory bodies such as the U.S. Food and Drug Administration (FDA) or the European Medicines Agency (EMA). Notable examples include Transpara, Koios DS, and ProFound AI, which have already been integrated into routine medical practice. Integration into clinical workflows also presents significant obstacles, as CAD systems must demonstrate interoperability with existing medical imaging infrastructures such as Picture Archiving and Communication Systems (PACS), while ensuring that radiologists are adequately trained in the use of AI-assisted tools. Moreover, ethical concerns related to patient privacy, data security, and informed consent must be carefully addressed to ensure trustworthy system development. These aspects underline that, beyond algorithmic performance, the successful integration of CAD systems into clinical practice depends on compliance with regulatory standards, extensive validation in real-world clinical settings, and the establishment of ethical and clinically acceptable frameworks.

Thus, future research should focus on developing standardized, publicly available, large-scale, annotated datasets from multiple institutions and imaging centers, which would significantly enhance the performance, reliability, and clinical applicability of CAD systems by capturing a broader range of anatomical, demographic, and acquisition-related variations. These datasets should also contain images from different imaging modalities for the same case, enabling the development of multi-modal CAD systems that could significantly advance breast cancer detection by providing a comprehensive analysis of imaging data. In addition, universally accepted benchmarks, performance metrics, and testing protocols should be established to allow researchers to evaluate the performance of implemented models under comparable conditions, ensuring consistency and generalizability. Likewise, the integration of explainable AI (XAI) approaches that can improve the interpretability of CAD systems should be prioritized, enhancing the level of trust and adoption by clinicians.

Future studies may examine the potential of modalities that have not been extensively employed in CAD systems, such as Thermography, MRI, PET, and MBI. Similarly, even though ANN, CNN, and U-Net architectures are widely used in current breast cancer diagnosis research, other DL models like GANs, AEs, and DBNs have been used to a limited extent and should be further explored. These types of networks fall under the category of unsupervised learning techniques, which avoid the need for labeled datasets. Finally, as indicated in a few reviews, future studies should focus on the development of CAD systems capable of integrating ML-extracted imaging features with clinical data, such as genetic and pathological information, in order to improve diagnostic accuracy.

## 9. Conclusions

This systematic review synthesized evidence from 48 systematic reviews, providing a comprehensive overview of CAD systems for breast cancer diagnosis. Our findings reveal that mammography remains the most widely adopted imaging modality, while DDSM, MIAS, and INBreast are the most frequently used datasets. However, multimodal imaging integration and clinical tasks beyond the detection and classification of breast lesions remain only partially investigated. Despite the promising results, current CAD systems face several critical limitations. The insufficient availability of large-scale, high-quality, and publicly annotated datasets hinders generalizability, while the limited interpretability of deep learning methods and their scarce clinical validation restricts their integration into real-world practice. Therefore, future research should focus on the development of explainable and transparent AI models, the creation of standardized multimodal and multi-institutional datasets, and the implementation of prospective clinical studies that will evaluate CAD performance in real-world clinical settings. By providing a thorough analysis of the current achievements and the remaining challenges, this systematic review can serve as a valuable resource for researchers and professionals in both medical and computer sciences, as well as provide future research directions.

## Figures and Tables

**Table 1:** Query formation.

Group A: Machine Learning-related keywords	Machine Learning AND Computer-Aided Diagnosis System
Group B: Medical-related keywords	Breast lesion AND Breast Tumor AND Breast cancer
Group C: Screening modalities related keywords	MRI OR Ultrasound OR Mammography OR Histopathological OR Thermography OR Digital Breast Tomosynthesis
Query	(Group A) AND (Group B) AND (Group C)

**Table 2:** List of Scopus journals and the corresponding number of papers.

Cancers (6)	*Ultrasonography* (1)
*Computers in Biology and Medicine* (4)	*Applied Artificial Intelligence* (1)
*Journal of Magnetic Resonance Imaging* (3)	*Seminars in Nuclear Medicine* (1)
*Diagnostics* (3)	*Open Life Sciences* (1)
*British Journal of Radiology* (2)	*Physica Medica* (1)
*Expert Systems with Applications* (2)	*Journal of Medical Imaging and Health Informatics* (1)
*Frontiers in Oncology* (2)	*BioMed Research International* (1)
*Tomography* (2)	*Current Oncology* (1)
*Computer Methods and Programs in Biomedicine* (2)	*Evolving Systems* (1)
*Saudi Medical Journal* (1)	*Current Medical Imaging* (1)
*Journal of International Medical Research* (1)	*Advanced Ultrasound in Diagnosis and Therapy* (1)
*Applied Sciences* (Switzerland) (1)	*EXCLI Journal* (1)
*Physics in Medicine and Biology* (1)	*Technology in Cancer Research and Treatment* (1)
*Advances in Distributed Computing and Artificial Intelligence Journal* (1)	*International Journal of Computing and Digital Systems* (1)
*International Journal of Emerging Technology and Advanced Engineering* (1)	*Wiley Interdisciplinary Reviews: Data Mining and Knowledge Discovery* (1)
*Archives of Computational Methods in Engineering* (1)	

**Table 3:** Comparison of breast cancer imaging modalities.

Imaging Modality	Principle	Radiation Exposure	Invasiveness	Suitable for	Advantages	Limitations
Mammography	2D imaging using low-intensity X-rays	Yes	Non-invasive	Routine breast cancer screening	- Widely available - Effective for detecting micro-calcifications - Cost-effective	- Low sensitivity in dense breasts - High false positive rate - Patient discomfort
DBT	3D imaging using multiple low-intensity X-ray projections	Yes	Non-invasive	Breast cancer detection in dense breasts	- Reduces tissue overlap - Able to detect small tumors - Good for dense breasts	- Higher radiation exposure and additional reading time compared to mammography - Expensive
Ultrasound	Image generation using high-frequency sound waves	No	Non-invasive	Detecting and characterizing cystic vs. solid masses	- Widely available - Cost effective - No radiation - Good for dense breasts - Fast acquisition	- Operator dependent - Low specificity
MRI	Generation of high-quality 3D images using strong magnetic fields	No	Non-invasive	- Breast cancer detection in high-risk patients or patients with dense breasts - Staging evaluation - Assist in treatment planning	- High sensitivity - No radiation - Good for dense breasts	- Expensive - Low specificity - Time consuming - Limited availability
Histopathology	Breast tissue biopsy analyzed under a microscope	No	Invasive (Biopsy required)	Definitive cancer diagnosis	- Gold standard for diagnosing malignancy - Provides tumor molecular insights	- Invasive (requires sample collection) - Time consuming
Thermography	Detects heat patterns associated with higher metabolic activity	No	Non-invasive	Early detection based on metabolic activity	- No radiation - Painless - Cost effective - Suitable for frequent monitoring	- Low specificity - Affected by external factors (room temperature)
Computed Tomography	Generation of high-resolution cross-sectional images using X-ray beams	Yes	Minimally invasive (requires contrast agent injection)	- Staging and metastasis evaluation - Assist in treatment planning	- Able to detect distant metastases - Less costly than MRI	- Radiation exposure - Low contrast - Not ideal for routine screening
PET	Visualize and identify changes in metabolic processes of breast tissue using radiotracers	Yes	Minimally invasive (requires radiotracer injection)	- Detect advanced breast cancer - Staging evaluation - Assessing cancer metastases and disease recurrence	- Functional imaging - Detects metastases effectively	- Radiation exposure - Expensive
Microwave Breast Imaging	Use low-power microwave signals to differentiate breast tissue based on dielectric properties	No	Non-invasive	Potential alternative for early cancer detection	- No radiation - Painless - Cost effective	- Lower resolution - Still under research

**Table 4:** Classification of CNNs based on the number of layers used.

Class	Number of Layers	Use Case	Examples
Shallow CNNs	2–10	Simple tasks, such as binary tumor classification (benign vs. malignant) on small datasets	LeNet-5, AlexNet
Moderately Deep CNNs	10–50	- Feature extraction from mid-sized image datasets - Multi-class tumor classification	VGG-16, VGG-19, GoogLeNet
Deep CNNs	50+	- Advanced segmentation and classification of breast lesions in large-scale datasets - Automated multi-modal fusion	ResNet, DenseNet, EfficientNet, DarkNet, ConvNeXt

## Data Availability

Data sharing is not applicable to this article as no new data were created or analyzed in this study. The raw data that support the findings of this study will be made available from the corresponding author upon reasonable request.

## Supplementary Materials

The following supporting information can be downloaded at: https://www.mdpi.com/article/10.3390/bioengineering12111160/s1, Table S1: Most common public datasets for different imaging modalities and their corresponding URL; Table S2: Names of the most common public datasets and their acronyms; Table S3: Machine Learning techniques used in the included reviews.

## Abbreviations

The following abbreviations are used in this manuscript:

ADC	Apparent Diffusion Coefficient
AEs	Autoencoders
AGs	Attention-based Generators
AI	Artificial Intelligence
ANN	Artificial Neural Network
AUC	Area Under Receiver Operating Characteristic Curve
BDT	Boosted Decision Tree
CAD	Computer-Aided Diagnosis
CC	Craniocaudal View
CNN	Convolutional Neural Network
CRF	Conditional Random Field
CT	Computed Tomography
DBN	Deep Belief Network
DBT	Digital Breast Tomosynthesis
DCE-MRI	Dynamic Contrast-Enhanced Magnetic Resonance Imaging
DCNN	Deep Convolutional Neural Network
DL	Deep Learning
DM	Digital Mammogram
DNN	Deep Neural Network
DQN	Deep Q-Network
DSC	Dice Similarity Coefficient
DT	Decision Tree
DTF	Decision Tree Forest
DWI-MRI	Diffusion-Weighted Magnetic Resonance Imaging
ELM	Extreme Learning Machine
EMA	European Medicines Agency
EUSOBI	European Society of Breast Imaging
FCM	Fuzzy C-Means Algorithm
FDA	U.S. Food and Drug Administration
FN	False Negative
FP	False Positive
GAN	Generative Adversarial Network
GLCM	Gray-Level Co-occurrence Matrix
GLRM	Gray-Level Run-Length Matrix
GMM	Gaussian Mixture Model
H&E	Hematoxylin and Eosin
IHC	Immunohistochemistry
IRT	Infrared Thermography
K-NN	K-Nearest Neighbor Algorithm
LDA	Linear Discriminant Analysis
LR	Linear Regression
MBI	Microwave Breast Imaging
ML	Machine Learning
MLO	Mediolateral Oblique View
MLP	Multilayer Perceptron
MRI	Magnetic Resonance Imaging
NAC	Neoadjuvant Chemotherapy
NB	Naïve Bayes Classifier
NFS	Neuro-Fuzzy System
OPF	Optimum-Path Forest
PACS	Picture Archiving and Communication Systems
PET	Positron Emission Tomography
PRISMA	Preferred Reporting Items for Systematic Reviews and Meta-Analyses
QDA	Quadratic Discriminant Analysis
RBF	Radial Basis Function Network
ReLU	Rectified Linear Unit Function
RF	Random Forest
RNN	Recurrent Neural Network
ROC	Receiver Operating Characteristic Curve
ROI	Region of Interest
SDT	Single Decision Tree
SegNet	Segmentation Network
SFM	Screen Film Mammogram
SOM	Self-Organizing Map
SVM	Support Vector Machine
TN	True Negative
TP	True Positive
UF-MRI	Ultrafast Breast Magnetic Resonance Imaging
US	Ultrasound
ViTs	Vision Transformers
WHO	World Health Organization
WNNs	Wavelet Neural Networks
WSI	Whole-Slide Image
XAI	Explainable AI
