# Supplementary material for: Application of CAD Systems in Breast Cancer Diagnosis Using Machine Learning Techniques: An Overview of Systematic Reviews
Source: Bioengineering (Basel). 2025 Oct 27;12(11):1160. doi: 10.3390/bioengineering12111160 (PMC12649147; doi:10.3390/bioengineering12111160)
Supplement: Supplementary file 1 [file bioengineering-12-01160-s001.zip › bioengineering-3900671-supplementary.pdf]

Application of CAD Systems in Breast Cancer Diagnosis Using Machine Learning Techniques: An Overview of Systematic Reviews

Theofilos Andreadis[1], Antonios Gasteratos[1], Ioannis Seimenis[2] and Dimitrios Koulouriotis[3]

[1]Department of Production and Management Engineering, Democritus University of Thrace, Xanthi, Greece
[2]School of Medicine, National and Kapodistrian University of Athens, Athens, Greece
[3]School of Mechanical Engineering, National Technical University of Athens, Athens, Greece

✉ Theofilos Andreadis
theofilos.andreadis@yahoo.gr

Table S1 Most common public datasets for different imaging modalities and their corresponding URL

Dataset	Imaging Modality	Origin	Image format	No. of cases	No. of Images	URL
DDSM	Mammography	USA	LJPEG	2.620	10.480	http://www.eng.usf.edu/cvprg/mammography/database.html
CBIS-DDSM	Mammography	USA	DICOM	1.566	10.239	https://www.cancerimagingarchive.net/collection/cbis-ddsm/
INBreast	Mammography	Portugal	DICOM	115	410	https://www.academicradiology.org/article/S1076-6332(11)00451-X/fulltext
MIAS	Mammography	UK	PGM	161	322	https://www.repository.cam.ac.uk/items/b6a97f0c-3b9b-40ad-8f18-3d121eef1459
Mini-MIAS	Mammography	UK	PGM	161	322	http://peipa.essex.ac.uk/info/mias.html
IRMA	Mammography	Germany	PGM	-	14.410	https://paperswithcode.com/dataset/irma
BancoWeb LAPIMO	Mammography	Brazil	TIFF	320	1.400	http://lapimo.sel.eesc.usp.br/bancoweb/english/index.php
OPTIMAM	Mammography	UK	DICOM	465.000	Nearly 7 million	https://www.cancerresearchhorizons.com/our-portfolio/our-licensing-opportunities/optimam-mammography-image-database-omi-db
BCDR	Mammography & Ultrasound	Spain & Portugal	TIFF	1.734	7.315	https://bcdr.eu/information/about
BUSI	Ultrasound	Egypt	PNG	600	780	https://www.sciencedirect.com/science/article/pii/S2352340919312181
UDIAT	Ultrasound	Spain	PNG	-	163	https://helward.mmu.ac.uk/STAFF/m.yap/dataset.php
BreaKHis	Histopathological	Brazil	PNG	82	7.909	https://web.inf.ufpr.br/vri/databases/breast-cancer-histopathological-database-breakhis/
Duke-Breast-Cancer-MRI	MRI	USA	DICOM	922	773.253	https://www.cancerimagingarchive.net/collection/duke-breast-cancer-mri/
RIDER Breast MRI	MRI	USA	DICOM	5	2.400	https://www.cancerimagingarchive.net/collection/rider-breast-mri/
DMR-IR	Thermography	Brazil	JPG	149	3.749	https://visual.ic.uff.br/dmi/
WBCD	Multivariate	USA	CSV	-	699	https://archive.ics.uci.edu/dataset/15/breast+cancer+wisconsin+original
WDBC	Multivariate	USA	CSV	-	569	https://archive.ics.uci.edu/dataset/17/breast+cancer+wisconsin+diagnostic

Table S2 Names of the most common public datasets and their acronyms

Name of database	Acronym
Digital Database for Screening Mammography	DDSM
Curated Breast Imaging Subset of Digital Database for Screening Mammography	CBIS-DDSM
Mammography Image Analysis Society	MIAS
Mini-Mammography Image Analysis Society	Mini-MIAS
Image Retrieval in Medical Applications	IRMA
Breast Cancer Digital Repository	BCDR
Breast Ultrasound Image Dataset	BUSI
UDIAT Diagnostic Center	UDIAT
Breast Cancer Histopathological Image Classification	BreaKHis

Reference Image Database to Evaluate Therapy Response	RIDER Breast MRI
Database For Mastology Research with Infrared Image	DMR-IR
Breast Cancer Wisconsin (Original)	WBCD
Breast Cancer Wisconsin (Diagnostic)	WDBC

Table S3 Machine Learning techniques used in the included reviews

Review	Authors, Publisher	Type of Learning	Techniques used
[66]	Wang L., Frontiers in Oncology (2024)	DL	CNN, DCNN, RNN, DBN, AEs, U-Net, GAN, AGs
[68]	Arun Kumar et al., Technology in Cancer Research and Treatment (2023)	ML and DL	RF, K-NN, SVM, NB, ANN, MLP, CNN, DCNN, RNN, DBN, AEs, GAN
[60]	Guo et al., Journal of International Medical Research (2024)	ML and DL	DT, RF, K-NN, SVM, LDA, NB, AdaBoost, XGBoost, LogitBoost, ANN, MLP, ELM, CNN, DCNN, DNN, WNN, AEs, U-Net
[69]	Loizidou et al., Tomography (2022)	ML and DL	SVM, LDA, ANN, CNN, RNN, GAN
[18]	Madani et al., Cancers (2022)	ML and DL	RF, SVM, CNN, DCNN, DBN, RNN, GAN, U-Net
[42]	Abhisheka et al., Archives of Computational Methods in Engineering (2023)	DL	CNN, DCNN, GAN, RNN
[15]	Ranjbarzadeh et al., Computers in Biology and Medicine (2023)	ML and DL	DT, RF, K-NN, SVM, NB, ANN, MLP, CNN, RNN, RBF, DBN, AEs, GAN
[65]	Jalloul et al., Diagnostics (2023)	ML and DL	DT, RF, K-NN, SVM, NB, FCM, ANN, MLP, CNN, RNN, DCNN, U-Net
[40]	Tariq et al., Expert Systems with Applications (2021)	ML and DL	DT, RF, K-NN, SVM, NB, LR, XGBoost, ANN, MLP, ELM, CNN, DCNN, AEs, U-Net
[70]	Kim et al., Ultrasonography (2021)	DL	CNN, U-Net
[71]	Sharma et al., Advances in Distributed Computing and Artificial Intelligence Journal (2024)	ML and DL	DT, RF, K-NN, SVM, LDA, QDA, ANN, ELM, CNN, DCNN, U-Net
[72]	Loizidou et al., Computers in Biology and Medicine (2023)	ML and DL	RF, K-NN, SVM, LDA, ANN, MLP, ELM, CNN, DNN
[73]	Balkenende et al., Seminars in Nuclear Medicine (2022)	DL	CNN, DCNN, GAN, DQN, U-Net
[74]	Guo et al., Open Life Sciences (2022)	ML and DL	SVM, ANN, WNN
[75]	Nemade et al., International Journal of Emerging Technology and Advanced Engineering (2022)	ML and DL	DT, RF, K-NN, SVM, NB, MLP, ELM, CNN, RNN
[76]	Wang et al., Advanced Ultrasound in Diagnosis and Therapy (2023)	DL	CNN, DCNN, RNN, GAN, ViTs, U-Net
[77]	Chan et al., British Journal of Radiology (2020)	ML and DL	SVM, LDA, CNN, DCNN, RNN, AEs, U-Net
[78]	Ammar et al., International Journal of Computing and Digital Systems (2024)	DL	MLP, CNN, DCNN, RNN, DBN, AEs, GAN
[79]	Jiménez-gaona et al., Applied Sciences (2020)	ML and DL	DT, RF, K-NN, SVM, NB, LDA, ANN, MLP, CNN, DCNN, DBN, RNN, WNN, AEs, GAN, U-Net
[80]	Zebari et al., Applied Artificial Intelligence (2021)	ML and DL	DT, RF, K-NN, SVM, NB, LDA, LR, AdaBoost, XGBoost, K-means, FCM, ANN, ELM, CNN, DCNN, SOM, DBN, NFS, U-Net, GAN, AGs
[61]	Basurto-Hurtado et al., Cancers (2022)	ML and DL	DT, RF, K-NN, SVM, AdaBoost, K-means, FCM, ANN, CNN, DNN, DBN, AEs
[81]	Reig et al., Journal of Magnetic Resonance Imaging (2020)	ML and DL	DT, RF, K-NN, SVM, NB, LDA, AdaBoost, XGBoost, ANN, CNN, DCNN, U-Net, V-Net
[82]	Sahu et al., Physica Medica (2023)	ML and DL	DT, RF, K-NN, SVM, NB, LR, K-meroids, K-means, FCM, Rough C-means, ANN, MLP, ELM, SOM, CNN, RNN, AEs, U-Net, GAN
[20]	Carriero et al., Diagnostics (2024)	DL	CNN, DCNN, ViTs, U-Net
[83]	Mahoro et al., Current Oncology (2022)	DL	ANN, DNN, CNN, DCNN, U-Net
[84]	Shah et al., Computers in Biology and Medicine (2022)	ML and DL	DT, RF, K-NN, SVM, NB, LDA, LR, ANN, DNN, CNN, DCNN, GAN, U-Net

[85]	Meyer-Base et al., Journal of Magnetic Resonance Imaging (2021)	ML and DL	DT, RF, K-NN, SVM, NB, LDA, ANN, MLP, CNN
[39]	Mridha et al., Cancers (2021)	DL	ANN, ELM, CNN, DBN, AEs, GAN
[86]	Pathak et al., Current Medical Imaging (2021)	ML and DL	DT, RF, K-NN, SVM, NB, LDA, K-means, ANN, MLP, CNN, DCNN, U-Net
[87]	Alsharif WM., Saudi Medical Journal (2023	DL	ANN, CNN, DCNN
[88]	Li et al., Physics in Medicine and Biology (2023)	ML and DL	RF, SVM, ANN, MLP, CNN, DCNN
[89]	Lei et al., Frontiers in Oncology (2021)	ML and DL	DT, RF, K-NN, SVM, NB, LDA, ANN, CNN, DCNN, U-Net
[16]	Houssein et al., Expert Systems with Applications (2021)	ML and DL	DT, RF, K-NN, SVM, NB, LDA, ANN, ELM, CNN, DBN, WNN, NFS, U-Net
[90]	Afrin et al., Cancers (2023)	ML and DL	RF, ANN, CNN, DCNN, GAN, U-Net, SegNet
[91]	Michael et al., BioMed Research International (2021)	ML and DL	DT, K-NN, SVM, FCM, ANN, MLP, ELM, CNN, CRF, U-Net, AGs, GAN
[92]	Al-Karawi et al., Tomography (2024)	ML and DL	DT, RF, K-NN, SVM, NB, FCM, ANN, MLP, CNN, DCNN, U-Net, AGs
[93]	Brunetti et al., Diagnostics (2023)	ML and DL	RF, K-NN, SVM, NB, LR, LDA, AdaBoost, XGBoost, ANN, MLP, CNN, DCNN
[94]	Yengec Tasdemir et al., Wiley Interdisciplinary Reviews: Data Mining and Knowledge Discovery (2020)	ML and DL	DT, RF, K-NN, SVM, LDA, ANN, MLP, CNN, DBN
[11]	Jalalian et al., EXCLI Journal (2017)	ML and DL	DT, K-NN, SVM, LDA, ANN, MLP, CNN, RNN, WNN, DBN
[95]	Hosni et al., Computer Methods and Programs in Biomedicine (2019)	ML and DL	DT, RF, K-NN, SVM, NB, ANN, MLP, DCNN
[19]	Yassin et al., Computer Methods and Programs in Biomedicine (2018)	ML and DL	DT, RF, K-NN, SVM, NB, LDA, QDA, LR, AdaBoost, GentleBoost, FCM, ANN, MLP, ELM, CNN, NFS
[96]	Burt et al., British Journal of Radiology (2018)	ML and DL	RF, SVM, ANN, CNN, DBN, WNN, AEs, U-Net
[97]	Nithya et al., Journal of Medical Imaging and Health Informatics (2015)	ML and DL	DT, K-NN, SVM, NB, LDA, ANN, MLP, RBF, NFS
[98]	Debelee et al., Evolving Systems (2020)	ML and DL	DT, RF, K-NN, SVM, LDA, ANN, CNN, RNN, DCNN, AEs, U-Net
[17]	Uwimana et al., Computers in Biology and Medicine (2025)	ML and DL	RF, K-NN, SVM, LDA, FCM, MLP, ELM, DNN, CNN, DCNN, U-Net
[99]	Chia et al., Cancers (2025)	ML and DL	SVM, XGBoost, ANN, DNN, CNN, DCNN, GAN
[100]	Jannatdoust et al., Journal of Magnetic Resonance Imaging (2025)	ML and DL	K-NN, SVM, K-means, FCM, ANN, CNN, DQN, U-Net, V-Net, W-Net
[43]	Liew et al., Cancers (2021)	ML and DL	DT, K-NN, SVM, NB, K-means, ANN, MLP, DNN, CNN, DCNN, RNN
